# *Rhizophagus irregularis* MUCL 41833 Improves Phosphorus Uptake and Water Use Efficiency in Maize Plants During Recovery From Drought Stress

**DOI:** 10.3389/fpls.2019.00897

**Published:** 2019-07-16

**Authors:** Olivia Le Pioufle, Matike Ganoudi, Maryline Calonne-Salmon, Fatma Ben Dhaou, Stéphane Declerck

**Affiliations:** ^1^Earth and Life Institute, Applied Microbiology, Mycology, Université catholique de Louvain, Louvain-la-Neuve, Belgium; ^2^Institut National de la Recherche Agronomique, Rabat, Morocco; ^3^Ecole Polytechnique de Sousse, Sousse, Tunisia

**Keywords:** maize, arbuscular mycorrhizal fungi, drought recovery, phosphorus uptake, leaf gas exchange, water use efficiency

## Abstract

Irregular precipitations are likely to affect maize production in the future. Arbuscular mycorrhizal fungi (AMF) have been reported to increase maize resistance to drought, but their role on the short-term inorganic phosphorus (Pi) uptake, leaf gas exchange parameters and water content during recovery after drought remains poorly understood. Here, we investigated these parameters in maize plants colonized or not by *Rhizophagus irregularis* MUCL 41833. The mycorrhizal (M) and non-mycorrhizal (NM) plants were grown for a 3-week period in a circulatory semi-hydroponic cultivation system and were submitted to well-, moderately-, or poorly-watered conditions (WW, MW, and PW, respectively), the two latter conditions corresponding to moderate and severe droughts. The plants were then watered at field capacity for 42 h with a Pi impoverished Hoagland nutrient solution and the dynamic of Pi depletion in the nutrient solution, corresponding to Pi uptake/immobilization by the maize-AMF associates, was evaluated at 0, 9, 21, and 42 h. The CO_2_ assimilation rate (A), stomatal conductance (g_s_), transpiration (E), and instantaneous water use efficiency (WUE_i_) were also assessed at 0 and 42 h of circulation. Plant biomass, plant water content, phosphorus concentration and content, and leaf relative water content were evaluated at harvest. During recovery, Pi uptake was significantly higher in M versus NM plants whatever the water regime (WR) applied before recovery. AMF did not affect leaf gas exchange parameters before recovery but modulated g_s_ and E, and improved WUE_i_ after 42 h of recovery. At harvest, no significant difference in dry biomass was found between M and NM plants but shoot fresh weight was significantly higher in M plants. This resulted in an increased shoot water content in M plants grown in the MW and PW treatments. Surprisingly, leaf relative water content was significantly lower in M plants when compared with NM plants. Finally, P content and concentration were significantly higher in roots but not in shoots of M plants. Our results suggested that AMF can play a role in drought resistance of maize plants by increasing the Pi uptake and WUE_i_ during recovery after drought stress.

## Introduction

Maize (*Zea mays* L.) is the third most consumed cereal worldwide, after rice, and wheat (Food and Agriculture Organization [FAO], 2013). It is considered a main staple food in Southern and Eastern Africa, Central America, and Mexico, playing an important role in food security (Ranum et al., 2014). The trend for more erratic rainfall in the next 50 years poses, however, a severe threat to maize production (Li et al., 2009; Challinor et al., 2014; Rosenzweig et al., 2014). Indeed, as a C_4_ plant, maize is known to have a higher water use efficiency than C_3_ plants such as wheat and barley (Akita and Moss, 1972). Yet, maize tend to have higher grain yield reduction under water shortage conditions and a higher drought sensitivity when compared to wheat (Daryanto et al., 2016). This could be explained partly by the drought avoidance strategy of maize plants that show decreased stomatal conductance, transpiration and eventually lower photosynthesis rate under lower soil water content (Sinclair et al., 1975).

The mechanisms involved in plant resistance to drought have been widely documented (see review by Fang and Xiong, 2015), while those involved during drought recovery have been less explored. A recent study conducted on maize indicated, however, that plant physiological responses differed during drought and recovery and that the recovery phase could play a more important role than previously believed (Chen et al., 2016). This corroborated the study of Hayano-Kanashiro et al. (2009) who observed that gene expression patterns between drought tolerant and drought sensitive maize genotypes differed more during recovery from drought than during drought. Similar findings were reported in drought tolerant cowpea genotypes (Anyia and Herzog, 2004; Rivas et al., 2016) pointing at the importance of studying plant recovery to improve adaptability of plants to water paucity.

A number of studies have demonstrated the role of soil microorganisms in increasing drought resistance/tolerance to drought stresses (Kim et al., 2012; Coleman-Derr and Tringe, 2014). Among these are the arbuscular mycorrhizal fungi (AMF). These soil microorganisms form symbiotic associations with an estimate of 72% of land plant species (Brundrett and Tedersoo, 2018) and have been suggested as promising root-associates for drought mitigation in plants (Boomsma and Vyn, 2008) via several mechanisms. For instance, improved cell turgor via osmotic adjustment in shoots (Wu and Xia, 2006), neutralization of reactive oxygen species in tissues via the synthesis of enzymes (e.g., superoxide dismutase) (Ruiz-Lozano et al., 1996), the increased access to soil pores inaccessible to roots or root hairs (Smith and Read, 2008) and transport of water via the extraradical mycelium to the root cells (Sánchez-Díaz and Honrubia, 1994; Ahmad Khan et al., 2003), the enhanced water uptake in plants by stimulating the growth of root hairs (Zou et al., 2017) and by increasing root hydraulic conductivity through modulation of root aquaporin expression (Bárzana et al., 2012; Quiroga et al., 2018) have been reported [for details see reviews of Lenoir et al. (2016) and Plouznikoff et al. (2016)]. Remarkably, in inorganic P (Pi) limiting soils, AMF can ameliorate P nutrition of plants by accessing to remote locations of Pi through their extraradical hyphae (Smith and Read, 2008) and it has been demonstrated that this improved Pi nutrition can maintain optimal growth and water relations and therefore increase plant resistance to drought (Nelsen and Safir, 1982; Fitter, 1988; Augé, 2001).

In contrast to the many studies conducted on the role of AMF in plant drought resistance (see above), the beneficial effects of these microorganisms during recovery from drought is much less investigated, especially in maize. An improved Pi nutrition was reported in AMF-colonized maize (Subramanian and Charest, 1997) and wheat (Bryla and Duniway, 1997) plants following drought recovery. However, it was not clear which from the Pi uptake pathways during recovery or during drought contributed the most to the improved P nutrition. Studies conducted by Levy and Krikun (1980) in citrus plants and by Goicoechea et al. (2004) in *Anthyllis cytisoides* revealed an improved water status in AMF-colonized plants together with an improved photosynthesis, leaf stomatal conductance and transpiration, during a 5 and 20 days recovery period, respectively. In maize, Subramanian and Charest (1997) indicated an accelerated recovery of leaf-turgidity in AMF-colonized plants. To understand how AMF may help plants to better recover from drought it could be important to examine and compare the short-term dynamics of Pi uptake and to analyze leaf gas exchanges in presence/absence of these root symbionts. Recently, a semi-hydroponic cultivation system developed by Colpaert et al. (1999) for pine associated to ectomycorrhizal fungi, was adapted by Garcés-Ruiz et al. (2017) to study the short-term dynamics of Pi uptake/mobilization by maize plants associated with AMF. The mycorrhizal maize plants were grown in containers on perlite and irrigated with a Hoagland Pi-impoverished nutrient solution circulating in a closed loop. The Pi uptake dynamics by the plant/AMF associates was deduced from the Pi depletion in the circulating nutrient solution.

In the present study, the semi-hydroponic cultivation system was used to assess the effect of AMF on the dynamics of Pi uptake and on leaf gas exchanges of maize plants during the first 42 h of recovery from drought. The plants inoculated with *R. irregularis* MUCL 41833 and their non-colonized counterparts were grown under well-, moderately-, or poorly-watered conditions for 3 weeks and then watered at field capacity for 42 h with a Pi-impoverished nutrient solution circulating in a close loop. The dynamics of Pi uptake was monitored at 0, 9, 21, and 42 h, and leaf gas exchanges measured at times 0 and 42 h after setup of the circulatory system. Finally, the plant biomass, P content and concentration, water content and leaf relative water content were assessed at the end of the experiment.

## Materials and Methods

### Biological Material

The arbuscular mycorrhizal fungus (AMF) *R. irregularis* (Błaszk., Wubet, Renker, and Buscot) C. Walker and A. Schüßler as [“irregulare”] MUCL 41833 was supplied by the Glomeromycota *in vitro* collection (GINCO)^1^ and grown *in vitro* on excised Ri T-DNA transformed roots of carrot (*Daucus carota* L.) as described in Cranenbrouck et al. (2005).

Seeds of maize (*Z. mays* cv. ES Ballade) were supplied by the Centre Indépendant de Promotion Fourragère (CIPF)^2^. They were surface-disinfected as described in Garcés-Ruiz et al. (2017) and placed on wet paper (N°1, Whatman, United Kingdom) in closed plastic containers (Aarts Plastics, Netherlands) in the dark at room temperature (∼20°C) for germination during 4 days.

### Preparation of the Maize Plantlets

Three 5 L pots containing a sterilized (121°C for 15 min) substrate composed of vermiculite (PullRhenen, Netherlands), sand (quartz N°4, Euroquartz, Belg) and lava stone (DCM, Belgium) (w:w:w, 1:1:1) were prepared to produce mycorrhizal (M) plants. In each pot, six maize seedlings were placed with their roots in contact with an approximate of 100 spores of an *in vitro* culture of *R. irregularis*. Three other pots containing a similar substrate but without AMF inoculum were used to produce non-mycorrhizal (NM) plants. All the pots were maintained 4 months under greenhouse conditions (21°C/18°C (day/night), a relative humidity (RH) varying from 40 to 70%, a photoperiod of 16 h day^–1^ and a photosynthetic photon flux (PPF) of 120 μmol m^–2^ s^–1^) to produce maize plants with strong root systems either colonized or not with the AMF. The shoots of the plants were then cut, and six new 3-days-old disinfected maize seedlings were planted in the pots containing or not the AMF extending from the roots of the cut maize plants. The plants were watered twice a week with 1400 ml of deionized water and received additionally 500 ml of Pi-impoverished modified Hoagland solution (Hoagland^lowPi^ – see Garcés-Ruiz et al. (2017) for chemical composition). The plants were kept in a grow chamber set at 21/21°C (day/night), a RH of 75%, a photoperiod of 16 h day^–1^ and a PPF of 300 μmol m^–2^ s^–1^.

### Experimental Set Up

After 3 weeks, the maize plants were gently removed from the pots and roots carefully rinsed from the substrate with deionized water. Three plants from the M and NM treatments were randomly selected and root colonization evaluated (see below). The other plants were transferred to the greenhouse into individual containers that consisted of 500 ml wash bottles filled with 25 g of dry perlite [see Garcés-Ruiz et al. (2017) for details]. The bottles were inverted [i.e., with neck below closed by a nylon mesh of 100 μm size pore (Prosep B.V.B.A., Belgium) to avoid substrate loss] and disposed in holes made in flex foam supports disposed on tables. Nine containers without plants were also included as non-vegetated (NV) controls. Every 2 days, all the plants and the NV control containers received the same volume (i.e., between 60 and 100 ml) of Hoagland^lowPi^ solution. Deionized water was further added until saturation of the substrate (estimated until the first drops of water were released from the opening at the bottom of the containers). After 24 days of acclimatization in the perlite, the M and NM maize plants had the same height (*P* = 0.923). They were separated in three homogenous groups and grown under three WR (water regime): well-watered (WW^M^ and WW^NM^), moderately-watered (MW^M^ and MW^NM^) or poorly-watered (PW^M^ and PW^NM^), the two later corresponding to moderate and severe drought conditions, respectively (see below for details). Six replicates were considered per treatment and three NV control containers were assigned to each WR. Treatments and controls were randomly distributed among five tables, with each treatment represented at least once on each table. Position of the containers were finally randomized on each table. The plants were subsequently grown under the three WR for a 3 week-period. Every 2 days, the plants in the WW treatments were supplied with deionized water to maintain saturation of the perlite (see details above), while in the MW and PW treatments, the plants received 60 and 30 ml of deionized water corresponding to 50 and 25% of the volume of water necessary to saturate the substrate (estimated to 120 ml deionized water, data not shown). After 1 week, the circulatory system was started a first time with Hoagland^lowPi^ solution for 42 h without any analysis. In the two following weeks, plants were watered as described above before setting the circulatory system a second time. During this second period, the MW and PW treatments induced drought stresses in the maize plants as noticed by the severe decrease in CO_2_ assimilation rate and the wilting of the leaves (data not shown). To summarize, the maize seeds were germinated for 3 days, then associated or not with the AMF in 5 L pots for 3 weeks (21 days). The M and NM plants were then transferred in the semi-hydroponic cultivation system and acclimatized for 24 days. Three water regimes (WW, MW, and PW) were then applied for 1 week and an additional 2-week period, each period being followed by 42 h of recovery. At harvest, the plants were grown in the semi-hydroponic cultivation system for 49 days and were 73 days old.

### Measurement of Short-Term Pi Depletion in the Hoagland^lowPi^ Solution Circulating Through the Containers Containing the M and NM Maize Plants and Non-vegetated Controls

**FIGURE 1:**
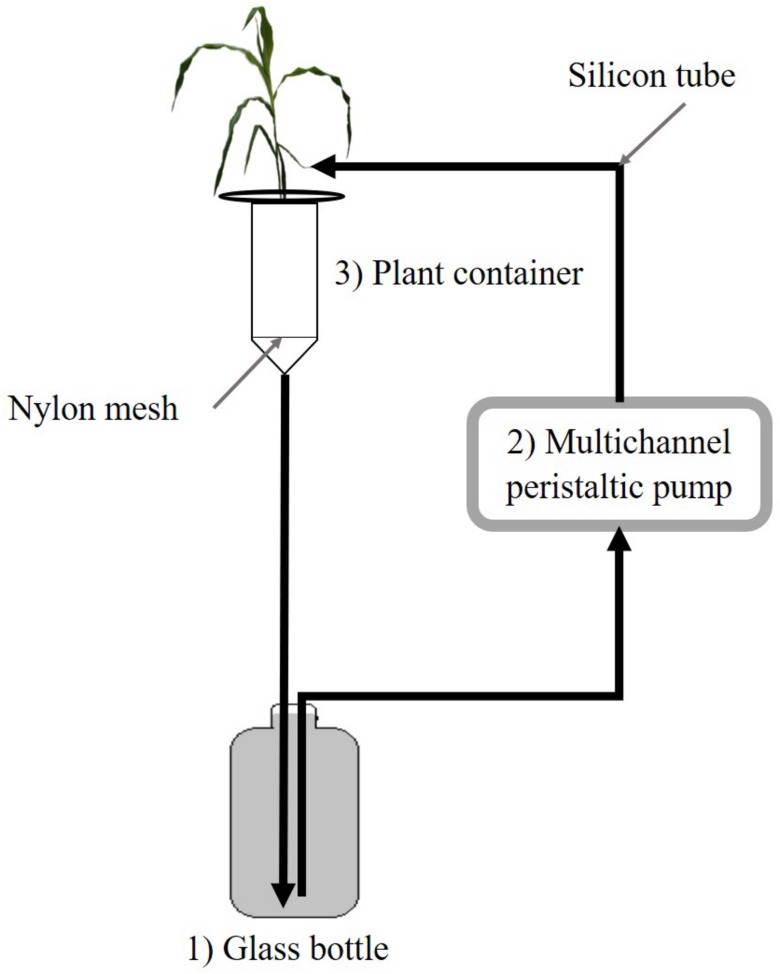
Schematic representation of the semi-hydroponic circulatory system [adapted from Garcés-Ruiz et al. (2017)]. Pi depletion is monitored during 42 h in the Hoagland^lowPi^ solution circulating through containers with mycorrhizal (M) or non-mycorrhizal (NM) maize plants. The nutrient solution in the glass bottle (1) is pumped with a peristaltic pump (2) to the upper part of the container (3) via silicon tubes. The solution percolates then through the plant container back into the glass bottle. Dark arrows indicate the direction of flow of the nutrient solution in the tubing.

The short-term depletion of Pi in the Hoagland^lowPi^ solution was monitored using a closed semi-hydroponic cultivation system as described by Garcés-Ruiz et al. (2017). Briefly, 1 L of Hoagland^lowPi^ solution contained in glass bottles circulated via peristaltic pumps through the containers with M or NM maize plants and returned to the bottles in a closed loop (see Figure 1). After a flushing step to equally saturate perlite in Hoagland^lowPi^ solution (Garcés-Ruiz et al., 2017), the Pi uptake by the plants and AMF was deduced from the Pi depletion in the circulating solution during 42 h. Initial Pi concentration in the nutrient solution was determined by sampling 15 ml of the solution from each bottle [considered as Time 0 h (T_0_)] before the start of the circulatory system. The circulatory system was then initiated at a rate of 7.4 ml min^–1^ and 15 ml of nutrient solution was collected at 9, 21, and 42 h. The samples were then stored at 4°C in the dark and Pi concentration subsequently measured by inductively coupled plasma atomic emission spectroscopy (ICP-AES) as described in Garcés-Ruiz et al. (2017). Data were then normalized according to the NV controls (Garcés-Ruiz et al., 2017). Finally, the data expressed in mg kg^–1^ were converted into % of Pi concentrations remaining in the nutrient solution relative to the initial concentration at T_0_ using the following formula:

(1) %ofPx=([Px]T+[PNV]T-0[PNV]T)/[Px]T×0100

where:

- [P] = Pi concentration in the solution
- x = sample
- NV = non-vegetated containers (i.e., perlite control) respective to the sample analyzed
- [PNV] = mean Pi concentration (three replicates) for the non-vegetated containers
- T = time considered (9, 21, or 42 h after the start of the circulatory system)
- T_0_ = 0 h before the start of the circulatory system.

### Leaf Gas Exchange Parameters in Maize Leaves

Net CO_2_ assimilation (A), stomatal conductance (g_s_), and transpiration rates (E) were measured in the morning (from 8 to 12 AM) before starting the circulatory system (considered as time = 0 h), and at the end of the circulation (at time = 42 h), using an InfraRed Gaz Analyzer (IRGA-LCi Photosynthesis system, ADC BioScientific Ltd.). For each plant, an average of three measures recorded from a 5.8 cm^2^ area on the 4 or 5^th^ leaf was considered for the analysis. Instantaneous water use efficiency was calculated using the following equation (Bacelar et al., 2012): WUE_i_ = A/E.

### Plant Harvest

At the end of the experiment, i.e., 49 days after transfer into the systems, the plants were harvested. The shoot and roots fresh weights (SFW and RFW, respectively) of each plant were evaluated and a leaf sample (approx. 1 × 7 cm) was cut from the 5^th^ leaf to evaluate leaf relative water contents (see below). Shoots and roots were oven-dried at 50°C for 7 days for evaluation of shoot and roots dry weights (SDW and RDW, respectively) and leaf samples were further used for the determination of the leaf relative water contents (RWC_L_-see below).

The root systems were subsequently separated in two parts to evaluate AMF root colonization and P concentration in tissues. After evaluation of dry weight, 500 mg of shoot and roots were ground separately for P analysis. Samples were digested following the procedure described in Garcés-Ruiz et al. (2017). Phosphorus concentration was quantified using ICP-AES.

RWC_L_ was determined at harvest from a sample of maize leaf (see above). After determination of the FW, the leaf sample was placed in a Petri dish containing water at 4°C in the dark for one night and turgid weight (TW) was estimated. Leaf samples were then oven-dried at 50°C for 4 h and dry weight (DW) was estimated. The RWC_L_ was calculated using the equation of Barrs and Weatherley (1962): RWC_L_ (%) = (FW − DW)/(TW − DW) × 100.

### AMF Root Colonization

Root colonization was estimated on three replicates from the M and NM plants at the time of transfer to the systems (i.e., 24 days old plants) and on each replicate of the six treatments at harvest (i.e., 49 days after transfer in to the systems – 73 days old plants). Roots were sampled, washed from the substrate, cut into small pieces and placed into 50 ml Falcon tubes (Sarstedt, Germany). Twenty-five ml of KOH 10% was added to the roots before incubation at 70°C in a water bath for 1 h. The KOH solution was removed and roots were washed with HCl 1%. The roots were further stained with ink 2% (Parker Blue Ink, United States) in HCl 1% (Walker, 2005) by placing the tubes at 70°C in a water bath for 1 h. The roots were rinsed and stored in deionized water before observation (Walker, 2005). Percentages of total (%TC), arbuscular (%AC), and vesicular (%VC) root colonization were estimated under a dissecting microscope (Olympus BH2–RFCA, Japan) at 10× magnification (McGonigle et al., 1990). At each intersection, the presence or absence of fungal structures was noted. Around 100 intersections were observed per plant.

### Statistical Analysis

Data analysis was performed with JMP 13.2 and SAS 9.4 statistical software (SAS Institute Inc., Cary, NC, United States) with a 5% α-threshold. Levene and Shapiro–Wilk tests were run prior to the statistical analysis to confirm homogeneity of variance and normality in the distribution, respectively. Phosphorus depletion in the Hoagland^lowPi^ nutrient solution was analyzed with a mixed model for repeated measurements, where “time” of sampling (i.e., 9, 21, and 42 h), “plant number” (from 1 to 36) and “table” (five different tables) were considered as random factors while “AMF” and “WR” (i.e., six conditions) were regarded as fixed factors. Plant biomass, tissue P content and concentration, and RWC_L_ were analyzed using a mixed model analysis with REML estimation where “AMF” and “WR” were regarded as fixed factors and “table” as random factor. Following significance of the factors, contrast analysis (*p* ≤ 0.05) was conducted to determine differences among groups. Data of g_s_ at time 0 h and data of g_s_ and E at time 42 h were submitted to a square root transformation to meet the assumption of the homogeneity of variance. Before and after circulation, A, g_s_ and WUE_i_ were analyzed using a mixed model with REML estimation followed by contrast analysis as described above. When a significant interaction between factors was found, a one-way ANOVA was run for each level of each factor “AMF” and “WR,” separately. Water contents was analyzed using Wilcoxon’s non-parametric comparison for each level of each factor “AMF” and “WR,” separately. Results of multiple pairwise comparisons were taking in account Bonferroni’s correction of *p*-values. Finally, root mycorrhizal colonization percentages were arcsine transformed and analyzed using a one-way ANOVA followed by a pairwise comparison using Tukey-HSD test. The raw data are provided as “Supplementary Material.”

## Results

### AMF Root Colonization

The root colonization parameters were measured at transfer of the maize plants into the containers and at harvest in the WW^M^, MW^M^ and PW^M^ treatments (Table 1). The %TC in the plants at transfer did not differ from the %TC in the plants of the WW^M^ and MW^M^ treatments at harvest, while it was significantly higher as compared to the plants in the PW^M^ treatment. Whatever the WR, no significant differences were observed in %AC between the plants at transfer and those at harvest. The %VC was significantly higher in the plants of the WW^M^ treatment as compared to the plants in the PW^M^ treatment but did not change significantly from the plants in the MW^M^ treatment and from the plants at transfer. No significant difference in %VC was noted between the plants in the MW^M^ and PW^M^ treatments as compared to the plants at transfer.

**TABLE 1:** Percentages of arbuscular- (%AC), vesicular- (%VC), and total colonization (%TC) of maize roots associated with the arbuscular mycorrhizal fungus *Rhizophagus irregularis* MUCL 41833 at transfer into the semi-hydroponic container system (after 21 days of pre-mycorrhization) and at harvest (in 73 days old plants that were grown for a 3-week-period under well- (WW), moderately- (MW), or poorly-watered (PW) conditions).

	**AC (%)**	**VC (%)**	**TC (%)**
At plant transfer	68 ± 6.4^a^	42 ± 6.3^ab^	97 ± 1.8^a^
Harvest time			
WW	69 ± 13.5^a^	47 ± 12^a^	77 ± 15ab
MW	51 ± 8.7^a^	37 ± 15ab	69 ± 18ab
PW	54 ± 13^a^	25 ± 11^b^	63 ± 19^b^

*Data were analyzed with a one-way ANOVA followed by pairwise comparison using Tukey-HSD test. Data in a column followed by similar letter do not differ significantly (p ≤ 0.05). Three replicates were considered at plant transfer into the containers and six per water regime (WW, MW, and PW) at harvest. Data are presented as mean ± SD.*

### Phosphorus Depletion in the Nutrient Solution

**FIGURE 2:**
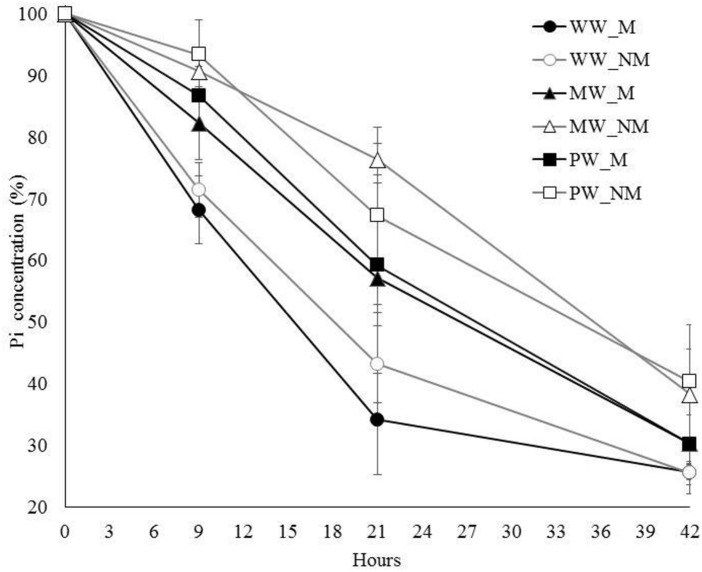
Dynamics of Pi depletion in the Hoagland^lowPi^ nutrient solution circulating in containers with mycorrhizal (M) and non-mycorrhizal (NM) maize plants grown under well- (WW), moderately- (MW), or poorly-watered (PW) conditions for a 3-week period before the start of the circulatory system. Percentages of Pi concentration remaining in the nutrient solution were expressed from Pi concentrations measured at times 9, 21, and 42 h relative to Pi concentrations measured at time 0 h. The AMF was *Rhizophagus irregularis* MUCL 41833. Data are presented as mean ± SD of six replicates per treatment.

The short-term dynamics of Pi depletion in the nutrient solution was assessed on maize plants that were grown for a 3-week-period under three contrasting WR (WW, MW, and PW) before watering them at field capacity for 42 h. Pi depletion (throughout the text expressed as relative percentage of Pi concentration in the medium or %Pi) was measured 0, 9, 21, and 42 h after setup of the circulatory system. Whatever the WR preceding circulation of the nutrient solution, a decrease was noticed in the %Pi in the nutrient solution between time 0 and 42 h (Figure 2), suggesting a concomitant Pi-uptake by the plant and AMF associates. Using the mixed model for repeated measures, the %Pi was affected by the factors AMF, WR, Time, and the interaction WR × Time (*P* < 0.0001), this later indicating that the impact of the WR significantly differed with time. Indeed, the %Pi was lower for the plants in the WW treatments when compared with those in the MW or PW treatments at any time but this difference was less significant at time 42 h than at times 9 and 21 h (at times 9 and 21 h, *P* < 0.0001 for plants in both MW and PW treatments when compared with plants in the WW treatments while at time 42 h, *P* = 0.007 and *P* = 0.004 for the plants in the MW and PW treatments, respectively, when compared with those in the WW treatments). No difference was found between the plants in the MW or PW treatments at times 9, 21 and 42 h (*P* = 0.425, *P* = 0.883, and *P* = 0.967, respectively). Conversely, the %Pi depletion was not affected by AMF × WR, AMF × Time, and AMF × WR × Time interactions (*P* > 0.05). This suggested that the effect of AMF on the %Pi depletion in the nutrient solution was effective whatever the WR or time considered. Interestingly, the plants in the MW^M^ and PW^M^ treatments took up proportionally more Pi (as compared to their respective NM controls) as compared to the plants in the WW^M^ treatment. Indeed, for the plants in the MW^M^ treatment, the decrease in %Pi was two times higher than for those in the MW^NM^ treatment at 9 and 21 h (i.e., 18 and 9% at 9 h and 43 and 23% at 21 h for the M and NM plants, respectively). For the plants in the PW^M^ treatment the decrease in %Pi as compared to the plants in the PW^NM^ treatment was 50 and 18% at times 9 and 21 h, respectively, while for the plants in the WW^M^ treatment, it was only 10 and 13% at times 9 and 21 h, respectively, as compared to those in the WW^NM^ treatment.

### Physiological Parameters

The values for A, g_s_, E, and WUE_i_ were analyzed in leaves before (i.e., Time 0) and after circulation of the nutrient solution (i.e., Time 42 h) (Table 2). At time 0 h, no significant effects of the factor AMF or the interaction AMF × WR were noticed for any of the parameters evaluated in the leaves. A significant effect of the factor WR was noticed on A, g_s_, and WUE_i_, but not on E. When compared with the plants in the WW treatments, the A, g_s_, and WUE_i_ significantly decreased in leaves by 58, 83, and 48%, respectively, in the plants of the MW treatments, and decreased by 25, 60, and 34%, respectively, in the plants of the PW treatments.

**TABLE 2:** Impact of different water regimes (WW, MW, and PW) and AMF colonization by *R. irregularis* MUCL 41833 on the net CO_2_ assimilation (A), stomatal conductance (g_*s*_), transpiration rate (E), and instantaneous water use efficiency (WUE_*i*_) measured in maize leaves at the start (0 h) and after 42 h of circulation of the nutrient solution. Mycorrhizal (M) and non-mycorrhizal (NM) maize plants were grown under well- (WW), moderately- (MW) or poorly-watered (PW) conditions for three weeks.

	**Time = 0 h**				**Time = 42 h**			
	**A (μmol CO_2_ m^–2^ s^–1^)**	**g_*s*_ (mmol m^–2^ s^–1^)**	**E (mmol H_2_O m^–2^ s^–1^)**	**WUE_*i*_ (μmol CO_2_ mol^–1^ H_2_O)**	**A (μmol CO_2_m^–2^ s^–1^)**	**g_*s*_ (mmol m^–2^ s^–1^)**	**E (mmol H_2_O m^–2^ s^–1^)**	**WUE_*i*_ (μmol CO_2_ mol^–1^ H_2_O)**
**Groups**								
M	0.8 ± 0.5	2.8 ± 4	0.2 ± 0.1	4.1 ± 2.4	1.4 ± 1.1	17 ± 22	0.5 ± 0.5b	3.0 ± 1.6a
NM	0.9 ± 0.4	2.2 ± 3.4	0.2 ± 0.1	5.4 ± 3.4	1.1 ± 1	23 ± 15	0.7 ± 0.4a	1.8 ± 1.2b

WW	1.2 ± 0.3α	4.7 ± 4.4α	0.2 ± 0.1	6.5 ± 3.7α	0.8 ± 0.6γ	14 ± 15	0.5 ± 0.4	2.4 ± 1.9
MW	0.5 ± 0.4γ	0.8 ± 2.1β	0.2 ± 0.1	3.4 ± 2.6β	1.3 ± 0.6β	17 ± 13	0.5 ± 0.3	2.7 ± 1.4
PW	0.9 ± 0.2β	1.9 ± 3.3β	0.2 ± 0.1	4.3 ± 1.5β	1.6 ± 1.5α	28 ± 25	0.8 ± 0.5	2.1 ± 1.3

WW^M^	1.2 ± 0.5	5 ± 4.6	0.2 ± 0.1	6.1 ± 2.6	0.8 ± 0.6	6.1±B*	0.3±0.2⁢B*	3.5 ± 1.9
WW^NM^	1.2 ± 0.2	4.4 ± 4.6	0.2 ± 0.1	7 ± 4.7	0.8 ± 0.8	22 ± 16^∗^*A*	0.7 ± 0.4^∗^*A*	1.2 ± 0.8

MW^M^	0.4 ± 0.3	1.1 ± 2.7	0.2 ± 0.1	2.1 ± 0.9	1.4 ± 0.8	12 ± 6.9*AB*	0.4±0.2⁢AB*	3.1 ± 1.4
MW^NM^	0.7 ± 0.5	0.6 ± 1.4	0.2 ± 0	4.7 ± 3.2	1.3 ± 0.5	22 ± 15 < *cps*:*it* > *A* < /*cps*:*it* >	0.7±0.4 < *cps:it > A < /cps:it >	2.4 ± 1.4

PW^M^	0.8 ± 0.2	2.2 ± 4.0	0.2 ± 0.1	4.1 ± 1.8	1.9 ± 1.6	32 ± 32A	0.8 ± 0.7A	2.5 ± 1.4
PW^*NM*^	0.9 ± 0.3	1.7 ± 2.8	0.2 ± 0.1	4.5 ± 1.3	1.3 ± 1.5	24 ± 11 < *cps*:*it* > *A* < /*cps*:*it* >	0.7 ± 0.4 < *cps*:*it* > *A* < /*cps*:*it* >	1.8 ± 1.2

**Effects**								
AMF	ns	ns	ns	ns	ns	ns	^∗^	^∗∗^
WR	^∗∗∗^	^∗^	ns	^∗^	^∗∗^	ns	ns	ns
AMF × WR	ns	ns	ns	ns	ns	^∗^	^∗^	ns
Table (% of the total effect)	0	18.2	4.45	16.6	60.62	32.96	45.3	21.16

*The effect of the fixed factors AMF colonization (AMF) and water regime (WR) as well as their interactions on plant physiological parameters were tested using the mixed model with REML analysis (p ≤ 0.05). Percentages of the effect due to the random factor Table are reported. When no significant interactions between fixed factors were found, significant differences among WR and between M and NM plants are indicated using contrast analysis. In the case of a significant interaction, the significant differences between the different levels of each treatment were tested separately using a one-way ANOVA followed by Tukey-HSD test (p ≤ 0.05). – Different lower-case letters indicate significant differences between M and NM plants according to the contrast analysis. – Different Greek letters indicate significant differences between WR according to the contrast analysis. – Different upper-case letters and different upper-case letters in italics indicate significant differences between WR inside each M and NM level, respectively. – Asterisks indicate significant differences between M and NM plants inside each WR level. Data are represented as mean ± SD of six replicates per treatment. For the P-values, ns = no significant difference, ^∗^p ≤ 0.05, ^∗∗^p ≤0.01, ^∗∗∗^p ≤ 0.001.*

At 42 h following circulation, the WUE_i_ measured in leaves was significantly higher in the M plants as compared to the NM ones (Table 2). The WR had an impact on A. Indeed, A significantly increased for the plants on the MW and PW treatments when compared to those in the WW treatments. The interaction AMF × WR significantly influenced g_s_ and E, suggesting that the impact of WR was dependent of the root colonization by the AMF and vice-versa. Indeed, whereas the parameters E and g_s_ remained similar in leaves of NM plants whatever the WR, their value increased in the leaves of the plants in the PW^M^ treatment as compared to those in the WW^M^ treatment. In addition, the plants in the WW^M^ treatment had a significant lower g_s_ than those in the WW^NM^ treatment, while under MW and PW conditions, no significant differences were noticed between M and NM plants. Indeed, the g_s_ measured in the leaves of the plants in the WW^M^ treatment was 72.3% lower as compared to those in the WW^NM^ treatment. Similarly, E significantly decreased by 57 and 42.9% in the leaves of the M plants as compared to NM plants, under WW and MW WR, respectively.

### Plant Growth Parameters

At the end of the experiment, plants were harvested and the FW and DW, the P concentration and content, as well as the WC and RWC_L_ were evaluated (Table 3). Whatever the WR, the SDW, P concentration and total P content in shoots did not differ significantly between the M and NM plants, while, the SFW was significantly higher in the M plants as compared to the NM ones. Conversely, the RWC_L_ was significantly higher in the NM plants as compared to the M plants. No significant difference was noticed in the shoot WC between the plants in the WW^M^ and WW^NM^ treatments, while under MW and PW conditions, a significant increase of shoot WC (by 2.9 and 2.6%, respectively) was noticed in the M plants as compared to the NM ones. Whatever the presence or absence of AMF within roots, the WR significantly impacted the SFW, SDW, and P concentration in shoots, but not the total P contents and RWC_L_. Indeed, the SFW and SDW significantly decreased in the plants of the MW and PW treatments. A significant lower P concentration was measured in the shoots of the plants in the WW treatments as compared to those in the MW or PW treatments. Nonetheless, when reported to P contents in shoots, no significant impact of WR was noticed. No significant interaction of WR × AMF was observed for any of the shoot parameters, demonstrating the absence of interactions between WR and presence/absence of AMF (results not presented).

**TABLE 3:** Impact of different water regimes (WW, MW, and PW) andAMF colonization by *R. irregularis* MUCL 41833 on freshweight (FW), dry weight (DW), water contents (WC), P concentration([P]), and P contents of shoots and roots and on leaf relative watercontents (RWC_L_) of maize at harvest time, after 73 days of growth. Mycorrhizal (M) and non-mycorrhizal (NM) maize plants were grown under well- (WW), moderately- (MW) or poorly-watered (PW) conditions for three weeks.

	**Shoots**						**Roots**				
	**FW (g)**	**DW (g)**	**WC (%)**	**RWC_*L*_ (%)**	**[P] (mg P g^–1^ of DW)**	**P contents (mg plant^–1^)**	**FW (g)**	**DW (g)**	**WC (%)**	**[P] (mg P g^–1^ of DW)**	**P contents (mg plant^–1^)**
**Groups**											
M	52 ± 7.7a	6.4 ± 2.3	88 ± 3.1a	75 ± 18b	1.5 ± 0.6	9 ± 2.6	23 ± 8.6	2.3 ± 1.0	89 ± 4	1.3 ± 0.2a	3 ± 1.3a
NM	46 ± 12b	6 ± 1.8	87 ± 2.b	90 ± 25a	1.5 ± 0.5	8.4 ± 2.6	23.4 ± 7.1	2.0 ± 0.6	91 ± 2	1 ± 0.2b	2 ± 0.5b

WW	59 ± 7.3α	8.1 ± 2.4α	86 ± 3.8	78 ± 23	1 ± 0.5γ	8 ± 3.3	30 ± 6.6α	2.8 ± 0.8α	90 ± 4	1 ± 0.6γ	2.9 ± 1.2α
MW	49 ± 7.3β	5.7 ± 1β	88 ± 2.3	84 ± 20	1.5 ± 0.3β	8.8 ± 2.4	23 ± 5.4β	2.1 ± 0.6β	91 ± 3	1.1 ± 0.3β	2.4 ± 1.1β
PW	40 ± 6.4γ	4.8 ± 0.6β	88 ± 1.6	88 ± 26	2 ± 0.4α	9.4 ± 2.0	17 ± 4.5γ	1.5 ± 0.6β	91 ± 3	1.3 ± 0.3α	1.9 ± 0.7γ

WW^M^	60 ± 5.3	8.9 ± 2.3	85 ± 3.7B	71 ± 21	0.9 ± 0.2	7.6 ± 2.5	30 ± 8.5	3.2 ± 0.9	89 ± 5	1.1 ± 0.1	3.7 ± 1.3
WW^NM^	59 ± 9.4	7.4 ± 2.5	87 ± 3.9 < *cps*:*it* > *A* < /*cps*:*it* >	83 ± 24	1.2 ± 0.6	8.4 ± 4.2	30 ± 4.8	2.4 ± 0.6	91 ± 2	0.9 ± 0.1	2.2 ± 0.4

MW^M^	52 ± 4	5.5 ± 1.1	89 ± 2.1⁢AB*	77 ± 14	1.7 ± 0.2	9.2 ± 2.5	22 ± 6.3	2.0 ± 0.8	90 ± 4	1.4 ± 0.3	2.8 ± 1.5
MW^NM^	46 ± 8.7	6 ± 0.9	86 ± 1.5 < *cps:it > A < /cps:it >	92 ± 22	1.4 ± 0.3	8.3 ± 2.4	23 ± 4.8	2.2 ± 0.4	91 ± 1	0.9 ± 0	2 ± 0.4

PW^M^	44 ± 4.5	4.9 ± 0.7	89 ± 0.7⁢A*	79 ± 21	2.1 ± 0.5	10 ± 2.5	16 ± 4.4	1.6 ± 0.6	90 ± 2	1.4 ± 0.1	2.2 ± 0.8
PW^NM^	35 ± 3.7	4.7 ± 0.4	86 ± 1.1 < *cps:it > A < /cps:it >	96 ± 30	1.8 ± 0.3	8.4 ± 0.9	17 ± 5.1	1.5 ± 0.6	91 ± 4	1.1 ± 0.3	1.5 ± 0.5

**Effects**											

AMF	^∗^	ns	^∗^	^∗^	ns	ns	ns	ns	ns	^∗∗∗^	^∗∗^
WR	^∗∗∗^	^∗∗∗^	ns	ns	^∗∗∗^	ns	^∗∗∗^	^∗∗∗^	ns	^∗∗∗^	^∗∗^
Table (% of the total effect)	0	0	na	8.7	7.6	0	3	0	na	6.5	5.6

*The effect of the fixed factors AMF colonization(AMF) and water regime (WR) as well as their interactions (result notpresented) on plant growth parameters was tested using mixed modelwith REML analysis (p≤0.05). Percentages of the effect due tothe random factor Table are reported. Water contents (WC) were analyzed by running Wilcoxon’s test on each factor separately and using Bonferroni’s correction for pairwise comparisons, without interaction analysis. For the [P], data in ppm were converted in mg g^−1^ of dry matter. – Different lower-case letters indicate significant differences between M and NM plants according to the contrast analyses. – Different Greek letters indicate significant differences between WR according to the contrast analysis – Different upper-case letters and lower-case letters in italic indicate significant differences between WR inside each M and NM level, respectively. – Asterisks indicate significant differences between M and NM treatments inside each WR level. Data are represented as mean ± SD of six replicates per treatment. For the P-values, ns = no significant difference, ^∗^p ≤ 0.05, ^∗∗^p ≤ 0.01, ^∗∗∗^p ≤ 0.001, na = test not applicable.*

In roots, no significant effect of the factor AMF was observed on the RFW and RDW whatever the WR, while P concentration and content were significantly higher in the M plants as compared to their respective NM controls (Table 3). These parameters were also significantly affected by the factor WR but not by the interaction AMF × WR. Indeed, the RFW and RDW as well as the P contents in roots decreased in the plants of the MW and PW treatments whatever the presence/absence of AMF. To the contrary, the P concentration in roots increased in the plants of the MW and PW treatments as compared to those in the WW treatments. No significant impact of AMF or WR could be observed on root WC.

## Discussion

Drought is a major threat to maize production and many strategies (e.g., irrigation, breeding) have been put in place to limit its impact. In maize, recovery after drought is poorly studied and the role played by AMF in this process is almost unknown. Here, we reported on the impact of *R. irregularis* MUCL 41833 on the dynamics of Pi uptake and on the modifications in leaf gas exchanges parameters [CO_2_ assimilation (A), stomatal conductance (g_s_), transpiration (E), and instantaneous water use efficiency (WUE_i_)] in maize plants that were grown under three contrasting WRs (i.e., well-, moderately- and poorly-watered, WW, MW, and PW, respectively) and subsequently watered at field capacity for 42 h. Plants were grown in a semi-hydroponic cultivation system to follow-up the depletion of Pi in the circulating nutrient solution. During the circulation, Pi uptake was increased in the AMF-colonized plants, whatever the WR. At initiation of circulation (time 0 h), A, g_s_, and WUE_i_ were impacted by the WR but not by AMF colonization, except for E which stayed unchanged whatever the condition. A, g_s_, and WUE_i_ were indeed lower in plants grown in the MW and PW treatments when compared with those in the WW treatments. After 42 h of recovery, A recorded in the plants in the MW and PW treatments exceeded those in the WW treatments, a significant interaction between AMF and WR was found for g_s_ and E, and WUE_i_ was improved in M plants when compared with NM plants whatever the WR. At harvest, the plants in the MW^M^ and PW^M^ treatments had higher shoot WC but also lower leaf RWC_L_ when compared to their non-mycorrhizal counterparts.

Total and arbuscular root colonization parameters evaluated at harvest were not impacted by the WR. This result is consistent with previous studies conducted on maize (Bárzana et al., 2014; Quiroga et al., 2017) and wheat under moderate and severe drought stress (Beltrano and Ronco, 2008) and suggested that the fungus was able to develop in the root system of plants under different WRs, even the most severe, as in our study. However, a significant decrease in %VC was found in the plants grown in the PW^M^ treatment when compared to those grown in the WW^M^ treatment. This corroborated the study of Martínez-García et al. (2012) that observed a decrease in vesicles but not in arbuscules in shrubs during seasonal drought. They suggested that under drought conditions, AMF would rather allocate energy to the production of arbuscules than to storage structures (i.e., vesicles) to maintain transfer of water and nutrients to the plant. Our results on Pi uptake during recovery (see below) tend to support this hypothesis with a proportionally higher uptake of Pi in the plants grown in the PW^M^ treatment versus those grown in the PW^NM^ treatment as compared to the plants grown in the WW^M^ treatment versus those grown in the WW^NM^ treatment, even if the data are non-significantly different.

The dynamics of Pi uptake/immobilization by the maize-AMF associates was measured indirectly by monitoring the % of Pi concentration remaining in the nutrient solution circulating through the plants. Whatever the presence/absence of AMF, the decrease in %Pi in the circulating solution in the plants of the WW treatments was fast from 0 to 21 h and slowed down onward 21 until 42 h. This dynamics is similar to the one encountered in studies using the same semi-hydroponic cultivation system with maize (Garcés-Ruiz et al., 2017) and medic (Calonne-Salmon et al., 2018) plants. A comparable, but less pronounced, decrease in %Pi was noticed in the M and NM plants grown under MW and PW treatments, although no slowdown was noticed after 21 h of circulation. Indeed, the Pi uptake in the plants of the MW and PW treatments was significantly lower as compared to those in the WW treatments whatever the time considered even if this difference was less significant at time 42 h. The lower Pi uptake could be explained by the significant lower root biomass of the plants in the MW and PW treatments as compared to those in the WW treatments, probably combined with a negative effect of drought on the plant and/or AMF physiology.

Whatever the WR, the Pi uptake by the plant/AMF associates during the 42 h circulation, increased significantly in the M versus NM plants. The absence of differences in root biomass between M and NM plants under similar WR, combined with the high levels of root colonization, suggested that the increased rate of Pi uptake in the M plants could be attributed to an increased acquisition of Pi by the extraradical mycelium extending in the substrate. However, the relative small volume of the container but also the continuous percolation of the nutrient solution through the substrate, might prevent the formation of Pi-depletion zones around roots and hyphae. Therefore, the increased Pi uptake in the M plants could probably result from an increased expression of fungal Pi transporters (Colpaert et al., 1999) rather than to an increased foraging by the extraradical hyphae. This result was also correlated with an improved Pi content in maize roots. However, shoot and root biomass as well as shoot P content and concentration at harvest were not improved in the M plants as compared to the NM plants under similar WR, as already reported in the study of Subramanian and Charest (1997) and Zhu et al. (2012) with maize. As suggested by Garcés-Ruiz et al. (2017), it is not excluded that Pi accumulated in the intraradical structures of the AMF (i.e., hyphae and vesicles) in the form of polyphosphate granules. Interestingly, similar shoot P contents under all WR were associated with significantly lower SDW in the plants of the MW and PW treatments as compared with those in the WW treatments. This suggested that water was the principal factor limiting plant growth. Pi accumulation in the intraradical structures of the fungus could thus been explained by the lower demand of Pi by the plant.

Leaf gas exchange parameters measured at the initiation of circulation (time 0 h) drastically differed after 42 h of circulation. A high increase of gs and E was noticed between time 0 and 42 h of circulation. As environmental conditions did not differ between both times (i.e., vapor pressure deficit, temperature, photosynthetically active radiation, not presented data), we hypothesized that these differences were related to the 42 h circulation. Interestingly, an increase of gs and E values in plants of the WW treatments was also noted between both times suggesting that perlite moisture at time 0 h was not optimal. It is indeed not excluded that a decrease in perlite moisture in the WW treatments had happened within 2 days, impacting gas exchange parameters event if no leaf rolling was observed in the plants in contrast to the MW and PW treatments.

At time 0 h, i.e., before plants received the nutrient solution, A, g_s_, and WUE_i_ in maize leaves were decreased in the plants of the MW and PW treatments as compared to those in the WW treatments. Conversely, after 42 h of circulation, g_s_ and WUE_i_ were not impacted anymore by the WR indicating that rehydration occurring through the circulation of the nutrient solution was sufficient to eliminate the differences between plants in the WW treatments and drought-stressed plants as already observed (Grzesiak et al., 2006; Hayano-Kanashiro et al., 2009; de Carvalho et al., 2011). In addition, the plants in the MW and PW treatments had significantly higher A values than those in the WW treatments at time 42 h and significantly increased shoot water contents at harvest. Increased CO_2_ assimilation has been considered a plant strategy to alleviate drought stress (Gale and Zeroni, 1985). This increased metabolism and water demand compared to the plants in the WW treatments suggest that plants in the MW and PW treatments tried to compensate the lack of growth and water supply during recovery.

At time 0 h, AMF did not improve leaf gas exchange parameters in plants that were grown under WW, MW, or PW conditions. Although various studies showed a beneficial effect of AMF on these parameters in well-watered or drought-stressed maize plants (Subramanian et al., 1995; Zhu et al., 2012; Augé et al., 2014), a few others reported no impact of AMF on g_s_ in drought-stressed maize plants (Quiroga et al., 2017) and on E parameters in well-watered maize plants (Subramanian and Charest, 1999). As suggested by Quiroga et al. (2017), the absence of effect could be due to the variability of response in different maize genotypes. After 42 h of circulation, AMF did not improve A but modulated E and g_s_ in function of the WR. Indeed, E and g_s_ were significantly lower in the plants of the WW^M^ treatment when compared to those in the MW^M^ and PW^M^ treatments. As photosynthesis was also significantly lower in the plants of the WW treatments when compared with those in the MW and PW treatments, it mechanically resulted in an improved WUE_i_ in the M plants comparatively to the NM plants.

An increased WC was observed in the plants of the MW^M^ and PW^M^ treatments during drought recovery which could be partly explained by the improvement of the WUE_i_ as observed in the plants at harvest. Other mechanisms such as improved root hydraulic properties during drought (Bárzana et al., 2012) or following recovery (Levy and Krikun, 1980) have also been proposed as possible reasons for increased WC in M plants under drought stressed conditions. Curiously, the higher WC in the plants of the MW^M^ and PW^M^ treatments was not associated with higher RWC_L_ measured from leaves. Indeed, RWC_L_ was significantly decreased by 15% in M plants when compared to NM plants. However, WC and RWC_L_ cannot strictly be compared as WC measurement is based on whole plant and takes in account water in leaves, stems, grains and flowers (if there are any), whereas RWC_L_ is measured from a small sample taken from the 4^th^ to 5^th^ leaf. This result seems also contradictory with previous studies which observed a higher RWC_L_ (Subramanian et al., 1997) and leaf moisture percentages (Zhao et al., 2015) in M maize plants during recovery. It has been shown that structure and physiology of maize leaves differed between mature and juvenile leaves (Bongard-Pierce et al., 1996). This difference in RWC_L_ could thus potentially be explained by the different maturity of the leaf measured in our study.

Meanwhile, the values of RWC_L_ measured in the plants of the WW treatments tended also to be lower than those in the plants in the MW and PW treatments. Thus, it seems that lower RWC_L_ values measured in plants of WW treatments as well as in M plants could reflect higher water binding properties of the plant tissues rather than a difference in the turgidity of the leaves. Plants tissues can indeed display different water binding properties depending on their structural and macromolecular composition. In wheat for example, it has been shown that leaves of drought tolerant cultivars had a higher affinity for strongly bound water when compared with drought sensitive ones (Rascio et al., 1999). This hypothesis is reinforced as the leave water contents (calculated without the contribution of the turgid weight) shows no significant differences among the treatments (not presented data).

In this study, we demonstrated a beneficial effect of AMF on the recovery of maize plants after a drought stress. An improved Pi uptake as well as an increased WUE_i_ and WC in shoots was noticed in the plant/AMF associates during the 42 h of recovery. Under a drought environment, where Pi is less available (Gahoonia et al., 1994) and precipitations are erratic, a more efficient Pi uptake by the fungus can help plants to mitigate the impact of drought stress and maintain growth (Singh et al., 1999) by improving net photosynthesis and water contents for instance (Garg et al., 2004). The ability of AMF to increase plant WUE_i_ during recovery from drought is of high agronomical importance as limited transpiration in maize plants is a major driver of yield performance in drought-susceptible environments (Messina et al., 2015). Therefore, future challenge could be to develop drought-adapted inbred lines highly responsive to AMF for faster recovery after drought stress.

## Data Availability

The raw data supporting the conclusions of this manuscript will be made available by the authors, without undue reservation, to any qualified researcher.

## Author Contributions

OLP contributed to the data collection, analysis, and interpretation, drafting of the manuscript, commentaries corrections, and final approval and agreement with all aspects of the work. MG contributed to the data collection, analysis, and interpretation, drafting of the results section and final approval and agreement with all aspects of the work. MC-S and SD contributed to the conception and design of the experiments, interpretation of the data, draft corrections, and final approval and agreement with all aspects of the work. FBD contributed to the data collection, and final approval and agreement with all aspects of the work.

## Conflict of Interest Statement

The authors declare that the research was conducted in the absence of any commercial or financial relationships that could be construed as a potential conflict of interest.

## Footnotes

1 http://www.mycorrhiza.be/ginco-bel

2 http://www.cipf.be/
